# Cold Storage Ordinance

**Published:** 1911-03

**Authors:** 


### Cold Storage Ordinance

THE ordinance committee of the board of aldermen of Buffalo has under consideration a new ordinance dealing with the cold storage problem. Owing to the proposed salary increases in the various departments which must be passed upon at once in order to prepare estimates for the coming fiscal year it is quite probable that the cold storage question will not be taken up for several weeks. The ordinance as drafted by the health department extends its power and jurisdiction in food inspection and the disposal of impure foods and authorises the health commissioner to destroy condemned food; it also permits the department inspectors to choose their own samples for examination and analysis. At present the inspectors must take the samples chosen by the dealers—a wholly farcical procedure. The new law provides a penalty of $250 to be recovered in civil actions and a fine of like amount in criminal cases, failure to pay which shall be followed by confinement in the penitentiary one day for each dollar of the fine not paid.

The proposed ordinance is most satisfactory and should not fail of favorable action and speedy passage.

---

A GENERAL order has been issued from the headquarters of the United States army looking to the general use of typhoid vaccine for the protection of troops. The experiments made by army surgeons at various points in the United States possessions have been so uniformly successful that the vaccine has been adopted as a routine procedure of administration. The vaccination is not compulsory. It is merely recommended.

The Court of Appeals of the state of New York has affirmed the conviction of Philip Friedman of Brooklyn, who was convicted of having in his possession and offering for sale 36 quarts of rotten eggs in a milk can. Friedman was sentenced to 60 days in the penitentiary on complaint of the health department of New York. This decision may have a deterrent effect in Buffalo.

---

In the Buffalo Medical Journal for February (*The Tribune*) appears the annual address of Dr. Grover Wende, president of the Medical Society of the County of Erie. The subject was "Mutual Relations of Physicians and Laymen," and in discussing it Dr. Wende said: "The fundamental fault of the layman, in his relation to the medical profession, is his failure to give proper consideration to the just claims of that profession, to appreciate its sincerity, to recognise its mission and to credit and confide in its worthy individuals. Until the true physician is appreciated and differentiated from the pretenders now flourishing by the credulity and gullibility of the public he will continue to suffer as he suffers now. Money getting is far from being the primary and ruling consideration with the conscientious physician."

---

The health of Buffalo for the month of January was generally good and compared favorably with other cities. There was a decided improvement over the December statistics. The diseases reported in January were as follows: mumps, 203 cases; chicken pox, 155; measles, 19; whooping cough, 91; typhoid, 33; diphtheria, 134; scaret fever, 163.

---

Dealing in eggs that are worse than bad (*The Tribune*) seems to be an expensive enterprise almost everywhere nowadays. New York's health authorities are active in prosecuting "rots and spots" cases, and *The Philadelphia Press* records a recent arrest in that city that cost the convicted merchant $250. "Incidentally," says *The Press,* "there was a moral in the announcement of Judge Ferguson that $50 was added to the fine because the defendant did not plead guilty. Apparently his honor believes that the man who will sell 'spots and rots' and then deny it in the face of positive evidence is himself a 'bad egg.' "

---

An association has been formed for the purpose of raising funds for a memorial for Dr. Willis G. Macdonald, late professor of clinical and abdominal surgery in Albany Medical College. Shortly before his death he had joined in a movement for new medical college buildings and more extensive laboratory facilities. A meeting was held in Albany, on February 3, 1911, where plans were discussed for raising the sum of $100,000. The memorial will probably take the form of a laboratory for Albany Medical College.

